# Transcriptomic correlates of nutritional manipulation in a facultatively social bee

**DOI:** 10.1242/jeb.250024

**Published:** 2025-04-16

**Authors:** Jesse L. Huisken, Sandra M. Rehan

**Affiliations:** Department of Biology, York University, Toronto, ON, Canada, M3J 1P3

**Keywords:** Developmental nutrition, Maternal manipulation, Caste development, Sibling care, Transcriptomics, Behavioural genetics

## Abstract

Subsocial behaviour in insects consists of extended parental care and may set the stage for the evolution of cooperation through manipulation of offspring. Manipulation of brood nutrition may produce differences in developmental or adult gene regulation, but it also produces smaller offspring which may be coerced into cooperation. The eastern small carpenter bee *Ceratina calcarata* frequently produces a smaller under-provisioned dwarf eldest daughter (DED). These DEDs are the only offspring to forage and feed siblings. To test whether nutritional manipulation of DEDs alters gene expression, inducing cooperative sibling care, we conducted a transcriptomic study, using whole heads, to assess differences in brain gene expression among naturally provisioned regular daughters and DEDs, experimentally under-provisioned regular daughters, and experimentally supplemented DEDs, prior to social interaction. Differences in gene expression were minimal among groups but were dramatic as a function of body size as a continuous variable, suggesting that differences in gene expression are more associated with absolute differences in body size, not discrete castes or order of eclosion. Enrichment for GO terms related to hormonal regulation in small bees points to hormonal regulation of transcription factors in behavioural differences that emerge in DEDs. Subordinate behaviours thus likely involve experience and social environment, though other developmental mechanisms, such as parental care, and later adult social interactions after eclosion, may act on differences in body size and gene expression to produce the distinct behaviour of DEDs.

## Teaser

**Highlighted Article:** Manipulation of offspring food provisions by mothers of the bee *Ceratina calcarata* reveals differences in gene expression associated with worker-like division of labour.

## INTRODUCTION

One of the long-standing goals of sociobiology is to understand the evolution of complex social behaviours and cooperation antecedent to the division of labour found in eusocial insects. One explanation proposes that parents may manipulate offspring to induce cooperation (Michener and Brothers, 1974). By limiting the opportunity for some offspring to establish their own nests, they are compelled to seek inclusive fitness opportunities by helping siblings (Lin and Michener, 1972). Such sibling care is commonly found in both vertebrates and invertebrates not regarded as eusocial, thus providing empirical evidence of rudimentary behaviours leading to eusociality, as remaining on the natal nest and mutual tolerance are requisite for social living (Briga et al., 2012; Stacey and Koenig, 1990).

An insight adapted from the field of evolutionary developmental biology proposes that parental manipulation of offspring during development may have long-lasting effects on adult phenotypes and account for the phenotypic plasticity found in eusocial insects (Amdam et al., 2004; West-Eberhard, 1987). Central to this explanation is the modularity of gene regulation, such that manipulation at critical developmental stages could produce the dramatic phenotypic differences found among social insect castes through alteration of molecular pathways and gene expression (Page and Amdam, 2007; Toth et al., 2007). Such potential modules are numerous, including those involved with reproductive regulation, parental care behaviour, and nutritional and metabolic systems (Alexander et al., 1991; Hunt and Nalepa, 1994; West-Eberhard, 1987). Evolutionary development thus predicts that distinct behavioural and physiological polyphenisms found in social insects will be associated with distinct gene expression profiles which may convergently recur throughout multiple origins of sociality (Rehan and Toth, 2015). There is a growing body of evidence from transcriptomic studies that such recurring modules exist in both obligately eusocial and facultatively social hymenopterans (Berens et al., 2015; Favreau et al., 2023; Rehan et al., 2018).

Within Hymenoptera, body size is largely determined during development through larval nutrition, making larval provisions a likely target of maternal manipulation (Hunt and Nalepa, 1994). Many obligately eusocial taxa possess physiologically distinct castes; larval nutrition reliably predicts caste development as observed in stingless bees, bumble bees and honey bees (Plowright and Pendrel, 1977; Quezada-Euán et al., 2011). Nutritional differences between honey bees are associated with distinct gene expression profiles leading to differences in brain development in adult castes (Moda et al., 2013). Nutrition is also a frequent determinant of social role in many facultatively social insects (Brand and Chapuisat, 2012; Packer and Knerer, 1985). In the social vespoid wasps, pre-imaginal caste determination is assumed to be primarily the product of differential nutrition (O'Donnell, 1998; Wheeler, 1986). In *Polistes* paper wasps, social role is frequently influenced by both larval and adult nutrition along with other factors and is associated with distinct gene expression profiles among castes (Daugherty et al., 2011; Jandt et al., 2017).

Nutrition is by no means the only factor involved in caste determination. In some cases, nutritional factors are not strictly predictive of social role and are confounded or mediated by other variables, with under-fed individuals being smaller or having lesser fat bodies (Karsai and Hunt, 2002). In sweat bees, nutrition leads to differences in size and/or ovarian development, which consequently influences the outcome of social conflicts in the nest (Eickwort, 1986; Greenberg and Buckle, 1981; Richards and Packer, 1994). In paper wasps, chemical signals of ovarian development interact with behaviourally established social hierarchies (Mitra and Gadagkar, 2012). This leaves the possibility that traits determined during development, including body size, ovary size and extent of fat bodies, are mediators of behaviourally determined social roles and are indirectly impacted by nutrition.

The forgoing systems contrast with many facultatively social species that may lack differences in body size and establish social roles behaviourally, through repeated aggressive interactions or through complex behavioural or chemical profiles that may be difficult to detect (Jandt et al., 2014; Sharma et al., 2022). For facultatively social systems, nutritional development and correlated body size may play a mediating role or may be entirely irrelevant. Size as a factor in solitary antecedents of eusocial lineages is not always clear, as evidenced by the solitary sister to the social sweat bees in the Halictinae, *Nomia melanderi*, where smaller bees are typically more aggressive (Smith et al., 2019). This suggests that sized-based hierarchies may not have existed in solitary species and may instead be derived characters (Smith et al., 2019). Understanding the role of nutritional development in both the proximate and ultimate evolutionary pathways of social behaviour is thus critical to identifying the broader patterns of social evolution across its many independent origins (Rehan and Toth, 2015).

Multiple experimental treatments of nutrition have elucidated its impact on social insect behaviour, but few of these studies include analyses of gene expression profiles (Gadagkar et al., 1991; Jandt et al., 2017). Those that do mainly consider obligately eusocial insects rather than those reflective of simple social systems and facultative sociality (Daugherty et al., 2011; Moda et al., 2013). Mothers of the facultatively social eastern small carpenter bee *Ceratina calcarata* frequently produce a smaller under-provisioned daughter, termed the dwarf eldest daughter (DED), that will forage to feed her adult siblings (Rehan and Richards, 2010a). *Ceratina calcarata* are univoltine, with a life cycle consisting of over-wintering in natal nests, mating and excavating new nests in pithy stems in the spring, provisioning brood cells and laying eggs in early summer, and have a second provisioning phase of feeding callow adult offspring in the late summer, during which time the DED becomes an active forager (Rehan and Richards, 2010a). The under-provisioned DEDs do not survive overwintering to breed the following spring, and thus rely on inclusive fitness through sibling care (Shell and Rehan, 2018).

As a model of social behaviour in insects, *C. calcarata* has provided insights into how brain gene expression may be associated with social role, age, social environment, maternal care and conflict (Rehan et al., 2014; Shell and Rehan, 2019). An outstanding question in this facultatively social bee is does nutritional manipulation constitute a developmental caste bias or is the marked difference in behaviour exhibited by DEDs mediated by well-documented aggressive behaviours between nestmates (Huisken et al., 2021; Rehan and Richards, 2013)? To determine how differences in nutrition may alter gene expression in the brain and potentially bias some individuals to perform co-operative tasks, we conducted a transcriptomic analysis of differences in gene expression between naturally provisioned, nutritionally deprived and supplemented brood prior to interaction with any adults. Brain gene expression profiles of callow adults were compared before any interactions between bees to determine how nutritional manipulation may bias worker-like daughters to perform co-operative tasks. It is expected that nutritionally deprived individuals will exhibit smaller body sizes and be biased to adopt a worker-like subordinate role and will exhibit significant differences in gene regulation prior to eclosion, some of which will overlap with gene expression patterns previously found in DEDs of this species (Lawson et al., 2017; Rehan et al., 2014; Shell and Rehan, 2019). This research provides valuable insights into the influence of nutritional manipulation and order of eclosion on establishing social hierarchies and producing worker-like cooperative behaviours. Our results are critical to identifying how cooperation and sibling care are established in facultatively social species that may be fundamental to understanding the evolution of obligate eusociality.

## MATERIALS AND METHODS

### Nest sampling and bee rearing

In April and May of 2021, wild sumac (*Rhus typhina*) and cut and dried raspberry (*Rubus idaeus*) branches at seven sites in the Greater Toronto Area were monitored for newly established *Ceratina calcarata* Robertson 1900 nests. Wild nests were opened weekly by splitting occupied stems longitudinally using a knife to establish seasonal progress of *C. calcarata* nesting. Beginning 7 June 2021, nests were collected before 09:00 h to ensure the mother could be collected along with her offspring. Nests were brought to the lab and opened using a knife. Foundresses were anaesthetized on ice and their head width, a proxy for overall size, was measured using a hand lens with an ocular micrometer before being flash frozen (Rehan and Richards, 2010b). Where present, eggs and small larva from brood cells one or three and their associated pollen balls were weighed using a Metler XPR2 and placed in ventilated PCR tubes. Brood and larvae from other cells or in later developmental stages were used in other experiments being conducted in the lab.

On the day of nest collection and dissection, pollen provisions were weighed, and treatment groups were manipulated using a spatula. Treatment groups from cell one received supplementary provisions through addition of approximately one-third of another pollen ball from within the nest; those in cell three had their provisions reduced by removing approximately one-third of their pollen ball. This created two nutritional groups, low or high nutrition, each with a natural control and an experimentally manipulated group. Larva and provisions were re-weighed after manipulation to confirm the desired reduction or increase in provisions and returned to PCR tubes, where they were incubated at room temperature.

Developing bees were monitored and developmental stage was recorded daily. On the day an individual became a callow adult, it was sexed and its head width was measured using a hand lens. Males were released, and daughters were briefly anaesthetized on ice while their head width was measured using a hand lens, and were then flash frozen in liquid nitrogen. To confirm expected differences in size head width between mothers and offspring, these were compared using two sample *t*-tests. Head width among phenotypes (mothers and daughters) and experimental groups (low and high nutrition) was compared using an ANOVA.

### Differential gene expression and co-expression of genes

Daughters were selected for RNA extraction by comparing their head widths with mothers' head widths to confirm expected category, resulting in a total of five DEDs, four regular daughters, five supplemented DEDs and five deprived regular daughters (*n*=19; Table S2). Extractions from whole heads were done using the Qiagen RNeasy Kit with the included standard protocol, and RNA samples were stored at 4°C. Library preparation and sequencing was done at Genome Québec using the Illumina NovaSeq 6000 targeting an average depth of 46 Mb reads per sample. Reads were aligned to the *C. calcarata* genome (NCBI Bioproject PRJNA791561) using STAR 2.6.1 (Dobin et al., 2013).

All data exploration, differential and gene co-expression analyses were performed in R 4.3.3 (http://www.R-project.org/). To explore overall differences in gene expression, we created a principal components analysis (PCA) using the vst and plotPCA functions. Differential gene expression analysis in DESeq2 (Love et al., 2014) compared gene expression among four phenotypes and experimental groups: (1) DEDs, (2) supplemented DEDs, (3) regular daughters and (4) deprived regular daughters; as well as gene expression regressed on all individual samples' head width, a proxy for overall body size (Rehan and Richards, 2010b). To find modules of co-expressed genes across each group, we used a weighted gene co-expression network analysis (WGCNA) with the WGCNA 1.72.5 R package, with a soft threshold of 16 and a cutoff of 80% (Langfelder and Horvath, 2008). To determine gene ontology (GO) term enrichment within groups and modules, we used topGO 2.54.0 using a GO annotation created using blast2GO on default settings (https://bioconductor.org/packages/topGO/; Götz et al., 2008).

## RESULTS

### Nutritional manipulation and rearing

**Fig. 1.**
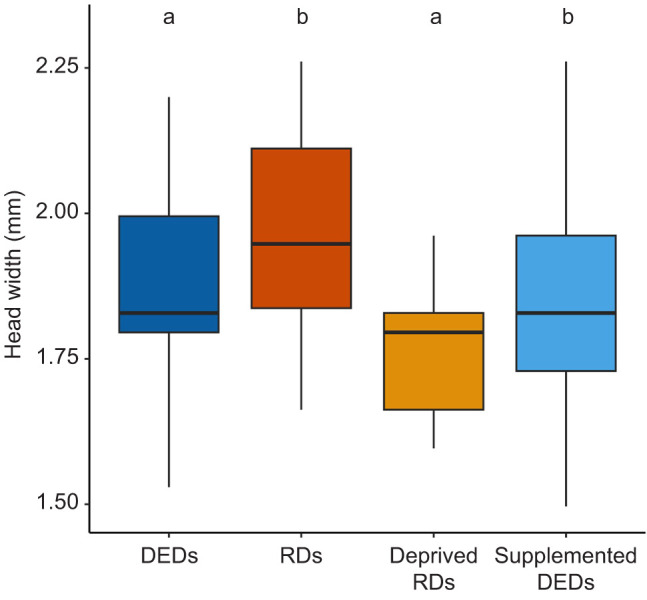
**Boxplot of head width at callow adult stages among each phenotype and experimental group.** DEDs, dwarf eldest daughters; RDs, regular daughters; deprived RDs, regular daughters on a reduced diet; supplemented DEDs, dwarf eldest daughters on a supplemented diet. Boxes represent quartiles around the median, error bars the standard error. Letters indicate statistically significant differences (*P*<0.05).

Of the 62 female bees surviving to callow adult stage, 7 DEDs, 11 regular daughters, 14 supplemented DEDs and 6 deprived regular daughters could be confirmed to belong to the expected phenotype or experimental group (Tables S1 and S2). Measurement of head width confirmed that mothers were significantly larger than DEDs and deprived regular daughters (DEDs: *t=*4.37, d.f.=13, *P=*0.0007; deprived regular daughters: *t=*5.07, d.f.=10, *P=*0.0005), but similar in size to regular daughters and supplemented DEDs (regular daughters: *t*=−0.41, d.f.=16, *P*=0.68; supplemented DEDs: *t=*0.31, d.f.=21, *P*=0.76). Size differences among phenotypes and experimental groups met the expected differences among categories (ANOVA: *F*=6.143, d.f.=3, *P*=0.002; Tukey test: regular daughter–DED, *P*=0.026; supplemented DED–DED, *P*=0.073; deprived regular daughter–DED, *P*=0.94; supplemented DED–regular daughter, *P*=0.92; deprived regular daughter–regular daughter, *P*=0.0075; deprived regular daughter–supplemented DED, *P*=0.021) (Fig. 1). In pairwise comparisons, regular daughters were found to be significantly larger than both deprived regular daughters and DEDs (deprived regular daughters: *t*=4.35, d.f.=15, *P*=0.0006; DEDs: *t*=−3.52, d.f.=16, *P*=0.003; Fig. 1), and DEDs were significantly smaller than supplemented DEDs (*t*=−2.98, d.f.=19; *P*=0.008; Fig. 1). Regular daughters and supplemented DEDs were of similar size, as were DEDs and deprived regular daughters (regular daughters–supplemented DEDs *t*=0.58, d.f.=22, *P*=0.57; DEDs–deprived regular daughters *t*=1.4345, d.f.=11, *P*=0.18; Fig. 1).

### Differential gene expression among groups

**Fig. 2.**
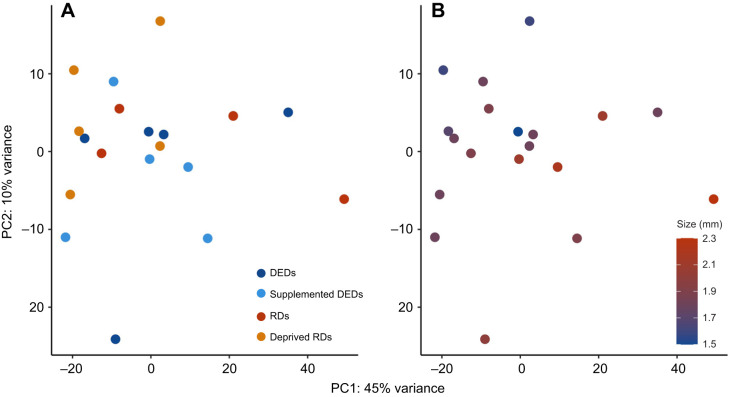
**Principal components analysis (PCA) of all genes detected in RNA-seq.** Points are labelled by group (A), and by head width (B). Combined PCs 1 and 2 represent a total of 55% of variation in gene expression. DEDs, dwarf eldest daughters; RDs, regular daughters; deprived RDs, regular daughters on a reduced diet; supplemented DEDs, dwarf eldest daughters on a supplemented diet.

**Fig. 3.**
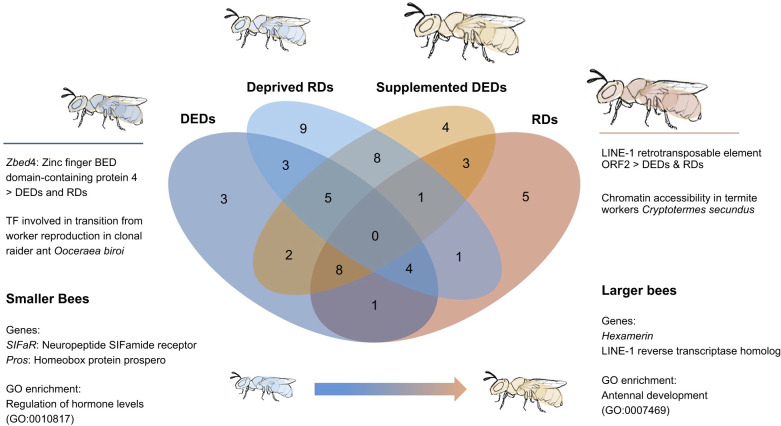
**Venn diagram of upregulated differentially expressed genes among the four groups, and between smaller and larger bees by absolute body size.** DEDs, dwarf eldest daughters; RDs, regular daughters; deprived RDs, regular daughters on a reduced diet; supplemented DEDs, dwarf eldest daughters on a supplemented diet. A full list of gene expression and GO terms is available in Table S4.

The results of our PCA for gene expression accounted for 45% and 10% in PCs 1 and 2, respectively, but showed little evidence of distinct gene expression patterns between samples of the same group (Fig. 2A); however, an overall trend of PC1 and PC2 being associated with differences in body size as a continuous variable was evident (Fig. 2B). A total of 129 differentially expressed genes (DEGs), representing 57 genes, were found when contrasting the four groups (Table S3). In total, 8 DEGs were uniquely upregulated in deprived regular daughters, the most uniquely upregulated genes in any group (Fig. 3).

Few upregulated genes were uniquely shared within expected nutritional groups; that is, between supplemented DEDs and regular daughters, and between deprived regular daughters and DEDs. Three were shared between DEDs and deprived regular daughters, and 4 between regular daughters and supplemented DEDs (Fig. 3). There were 12 upregulated DEGs found in low nutrition groups, i.e. DEDs and deprived regular daughters, including 8 proteins of unknown function, THAP domain containing protein 1 (*thap1*) and 3 genes derived from transposable elements (Fig. 3; Table S3). Across each comparison, the most differentially expressed genes included primarily proteins of unknown function and retrovirus related polyproteins (Table 1). Among the most significantly enriched GO terms for DEDs were peptide hormone processing (GO:0016486) and response to nutrient (GO:0007584). Regular daughters were enriched for post-embryonic body morphogenesis (GO:0040032), and many genes associated with female development, including intracellular estrogen receptor signaling, uterus and vaginal development (GO:0030520, GO:0060065, GO:0060068). WGCNA found that only two modules (modules 1 and 2) of co-expressed genes were statistically supported (*P*<0.05). These were both positively associated with regular daughters (*R*^2^=0.46 for both). Module 1 included 438 co-expressed genes, and module 2 included 325 (Table S6). Module 1 was enriched for GO terms including regulation of circadian rhythm (GO:0042752) and olfactory behaviour (GO:0042048). Module 2 was enriched for gravitaxis (GO:0042332), regulation of Ras protein signal transduction (GO:0046578) and regulation of hippo signaling (GO:0035330; Table S7).

**Table 1.** The five most highly differentially expressed genes across each contrast

Comparison	Most highly expressed genes	
DEDs>supplemented DEDs	Ccalc.v3.15354	Similar to LINE-1 retrotransposable element ORF2 protein
	Ccalc.v3.04114	Protein of unknown function
	Ccalc.v3.16847	Similar to Retrovirus-related Pol polyprotein from transposon TNT 1-94
	Ccalc.v3.17550	Protein of unknown function
	Ccalc.v3.17549	Protein of unknown function
DEDs>RDs	Ccalc.v3.23894	Similar to Retrovirus-related Pol polyprotein from transposon TNT 1-94
	Ccalc.v3.16563	Protein of unknown function
	Ccalc.v3.18497	Similar to X-element\ORF2: Probable RNA-directed DNA polymerase from transposon X-element
	Ccalc.v3.23682	Protein of unknown function
	Ccalc.v3.13866	Protein of unknown function
DEDs>supplemented RDs	Ccalc.v3.11299	Similar to Retrovirus-related Pol polyprotein from transposon TNT 1-94
	Ccalc.v3.19128	Protein of unknown function
	Ccalc.v3.22702	Protein of unknown function
	Ccalc.v3.02541	Protein of unknown function
	Ccalc.v3.15354	Similar to LINE-1 retrotransposable element ORF2 protein
RDs>supplemented DEDs	Ccalc.v3.20118	Similar to pol: Retrovirus-related Pol polyprotein from transposon 17.6
	Ccalc.v3.17550	Protein of unknown function
	Ccalc.v3.15354	Similar to LINE-1 retrotransposable element ORF2 protein
	Ccalc.v3.04114	Protein of unknown function
	Ccalc.v3.17549	Protein of unknown function
RDs>deprived RDs	Ccalc.v3.22702	Protein of unknown function
	Ccalc.v3.19128	Protein of unknown function
	Ccalc.v3.22127	Protein of unknown function
	Ccalc.v3.15354	Similar to LINE-1 retrotransposable element ORF2 protein
	Ccalc.v3.16953	Protein of unknown function
Deprived RDs>supplemented DEDs	Ccalc.v3.21178	Protein of unknown function
	Ccalc.v3.17550	Protein of unknown function
	Ccalc.v3.20118	Similar to pol: Retrovirus-related Pol polyprotein from transposon 17.6
	Ccalc.v3.16847	Similar to Retrovirus-related Pol polyprotein from transposon TNT 1-94
	Ccalc.v3.04114	Protein of unknown function

DEDs, dwarf eldest daughters; RDs, regular daughters; deprived RDs, regular daughters on a reduced diet; supplemented DED, dwarf eldest daughters on a supplemented diet. Groups in which the gene is upregulated are shown with greater than symbol (‘>’).

Twelve upregulated DEGs were also shared between supplemented nutritional groups including proteins of unknown function. A single gene was shared between these groups, Zinc finger BED domain-containing protein 4 (*Zbed4*). Supplemented DEDs were enriched for GO terms for body morphogenesis (GO:0010171) and tissue remodelling (GO:0048771). Deprived regular daughters were enriched for neurotransmitter uptake (GO:0001504) and tryptophan transport (GO:0015827). Across the four experimental treatments, DEGs showed some similarity between nutritional groups, i.e. between DEDs and deprived regular daughters (17 DEGs; Table S3) and between regular daughters and supplemented DEDs (65 DEGs; Table S3; Fig. 3). However, upregulated genes uniquely shared between control and treatment groups were few, with DEDs and supplemented DEDs sharing only two DEGs, and regular and deprived regular daughters sharing only one DEG (Fig. 3). There were eight uniquely shared upregulated genes between the two experimental groups or high and low nutrition (Fig. 3; Table S3).

### Differential gene expression by absolute body size

Gene expression was mostly associated with absolute body size, with 258 DEGs associated with increasing body size and 282 with decreasing size (Table S4). Among the most highly expressed genes associated with larger individuals were LINE-1 reverse transcriptase homolog, and four proteins of unknown function (Fig. 3; Table S4). Among the genes most highly expressed in small bees were genes encoding protein asteroid (*Ast*), neuropeptide SIFamide receptor (*SIFaR*), homeobox protein prospero (*pros*), zinc finger protein jing homolog (*jing*), enteropeptidase (*TMPRSS15*) and nine proteins of unknown function (Table S4). Genes most highly expressed in large bees included *hexamerin*, LINE-1 reverse transcriptase homolog, venom serine protease inhibitor, lipase 1 (*lip1*) and four proteins of unknown function (Table S4). A complete list of enriched GO terms is available in Table S5. Enriched GO terms for large bees included post-embryonic body morphogenesis (GO:0040032), antennal development (GO:0007469) and sensory perception of mechanical stimulation (GO:0050954). Enriched GO terms associated with smaller bees included zinc ion homeostasis (GO:0055069) and regulation of hormone levels (GO:0010817).

## DISCUSSION

By experimentally manipulating larval nutrition in a facultatively social bee, we were able to disentangle nutrition-related gene expression from other factors that may influence social phenotypes, including learning and memory, social environment, age of eclosion and brood cell position in nest. We tested whether nutritional manipulations act as a form of developmental bias, priming smaller DEDs to cooperatively feed their larger siblings. In doing this, we quantified brain gene expression of naive adult bees prior to any social interactions or learning, examining this in two ways: firstly, by considering differences among DEDs and regular daughters, and nutritionally manipulated relatively smaller and larger bees; and secondly, by quantifying differences in gene expression associated with absolute differences in body size; that is, separate from their relative classification as DEGs or regular daughters.

### Gene expression and nutrition

Our experiment found limited differences in gene expression associated with the distinct social and developmental categories of regular daughters and DEDs, and that few of these differences involved genes known to be related to social behaviour in the social insect literature. Differentially expressed genes shared between experimental and control treatments of larger or smaller bees, which are thus expected to stem exclusively from nutrition, were limited. Moreover, few of these DEGs overlap with those found in either DEDs or regular daughters in previous studies of this species (Huisken and Rehan, 2023; Rehan et al., 2014; Shell and Rehan, 2019).

Among the DEGs upregulated in both DEDs and deprived regular daughters known to play a role in social insect behaviour, *Zbed4* has previously been found to act as a hub gene in a gene co-expression module associated with *C. calcarata* regular daughters (Huisken and Rehan, 2023). Also related to social insect behaviour are several upregulated retrovirus-related polyproteins (Table S3). Similar retrovirus-related proteins have been found to be upregulated in polygynous colonies of the raider ant *Solenopsis invicta*, making them interesting targets for future genomic and functional work dissecting their role in social insect behaviour (Nipitwattanaphon et al., 2013). More generally, retrotransposons are thought to be an important source of novelty in gene expression, perhaps allowing for phenotypic plasticity through alterations of regulatory regions and transcription factors (Herpin et al., 2010; Huang et al., 2008; Lerat and Sémon, 2007). The few upregulated genes uniquely shared between the two experimental groups, deprived or supplemented, suggests only a limited effect of experimental nutritional manipulation itself.

Previous results have shown that nutritional deprivation of *C. calcarata* brood results in worker-like reduced aggressiveness even in the first social encounters of naive bees (Lawson et al., 2017; Rehan and Richards, 2013). Nutritional deprivation may also subtly alter overall avoidant behaviours within colonies of *C. calcarata* (Huisken and Rehan, 2022)*.* Given the few differences in gene expression found among discrete groups in our study, subordinate behaviours likely involve experience acquired during conflict (Huisken and Rehan, 2023; Mikát et al., 2017). Experience has been found to play a role in *C. calcarata* social conflict, with body size of the individual generally predicting outcomes in first encounters of naive bees but learned experiences predominating in subsequent ones (Withee and Rehan, 2016). Such changes in self-assessment during conflict are commonly found in solitary insects and vertebrates (Mesterton-Gibbons et al., 1996), and social hierarchies in primitively eusocial Hymenoptera are frequently flexible and established through repeated social interactions rather than fixed developmental features (Jandt et al., 2014; Unnikrishnan and Gadagkar, 2017).

The limited differences in gene expression found from nutritional development in our study contrast with significant changes in gene expression associated with social status, conflict and social environment in *C. calcarata* (Huisken and Rehan, 2023; Withee and Rehan, 2017). For instance, as many as 457 genes may be differentially expressed over the course of just two agonistic interactions, including many genes with known functions in synaptic growth, learning and memory (Withee and Rehan, 2017). Taken together with previous results, our study suggests that the limited differential gene expression found between discrete groups is not comparable to the dramatic changes in gene expression found in adults after social interactions with other conspecifics or nestmates. It is thus unlikely that nutrition alone plays a strong direct role in biasing DEDs or regular daughters to adopt their distinctive behavioural profiles.

### Gene expression and intrinsic size

In contrast to the limited differences between discrete DED and regular daughter groups, differences between large and small bees in terms of absolute body size were considerable, including some highly expressed genes and GO enrichment terms known to be involved in social insect behaviour. Genes associated with larger bees include *hexamerin*, also known to be involved in dominance in the facultatively eusocial bee *Euglossa dilemma*, and in the regulation of juvenile hormone in ants and termites (Hawkings et al., 2019; Saleh and Ramírez, 2019; Zhou et al., 2007). Juvenile hormone, a key gonadotropin in insects, has been found to play a role in caste differentiation in many social insects, including several bee species (reviewed in Bloch et al., 2002). Small bees showed high levels of gene expression of the key transcription factors *pros* and zinc finger protein *jing*. The gene *pros* encodes a transcription factor essential to nervous system development (Demidenko et al., 2001), and similar transcription factors, including *hairy*, are linked to genes upregulated in solitary females of facultatively social *Ceratina australensis* (Rehan et al., 2018). The presence of enriched GO terms related to hormonal regulation in small bees also suggests a primary role for hormonal regulation of transcription factors in setting the stage for behavioural differences to emerge in smaller bees.

In keeping with experiential factors operating in rudimentary forms of social life found in *C. calcarata*, social roles remain somewhat flexible in this species, with regular daughters sometimes adopting foraging roles (Mikát et al., 2017). Social environment may also dramatically increase the frequency of social interactions and alter levels of tolerance and aggression within nests (Huisken et al., 2021). Differences in behaviour may also result from nutritional intake directly altering brain chemistry (Sasaki et al., 2021). While high levels of dopamine expression in *Apis mellifera* queens are associated with greater expression of genes involved in dopamine synthesis, in *Bombus ignitus*, higher dopamine in queens instead stems from higher quantities of the dopamine precursors tyrosine and tryptophan found in the queen's diet (Sasaki et al., 2018). Thus, conserved physiological differences involved in social behaviour may result from diet in one social species but be rooted in endogenous production in another.

While discrete nutrition and body size differences are regarded as a hallmark of division of labour and caste in obligately eusocial insects (Molet et al., 2012; Trible and Kronauer, 2017), there is a growing appreciation that even obligately eusocial insect colonies may include individuals that are intermediate between castes or that possess mosaic phenotypes, likely resulting from the rearrangement or resynchronization of developmental modules (Abouheif, 2021; Molet et al., 2012). For instance, at the individual level, *A. mellifera* workers exist on a spectrum between nurse and forager behaviours, depending on age and associated gene expression (Johnson and Frost, 2012). Such phenotypic spectrums are also found in numerous ant species but were until recently generally recognized only from their most conspicuous examples and considered anomalies (Khalife et al., 2024; Londe et al., 2015; Plateaux, 1970). How gene expression is regulated across mosaics or spectrums of physiological and behavioural polymorphism is understudied in social insects.

Our results suggest that unlike obligately eusocial insect behaviour, where development is the major direct determinate of social phenotypes, developmental outcomes determining size are indirect factors influencing social behaviour in *C. calcarata* through experience, memory and learning acquired during conflicts (Withee and Rehan, 2016). The differences in gene expression and regulation found in different behavioural states in this species are thus largely established post-eclosion, through factors such as social environment and position in social hierarchy. Other developmental factors apart from nutrition may still be involved in the differentiation of DEDs and regular daughters (Withee and Rehan, 2017). Interactions with developing larvae and pupae, including chemical and mechanical manipulation, are all known to influence future social roles in social insects (Matsuura et al., 2010; Mignini and Lorenzi, 2015; Rajakumar et al., 2018). Previous studies have suggested that in *C. calcarata*, maternal care during development is associated with differences in gene expression, methylation and microbiome, both during development and in adults, and these are thus possible determinates of social behaviour (Arsenault et al., 2018; Chau et al., 2023). Future studies combining nutrition, microbiome and social environment analyses are needed to further assess the relative roles of order or eclosion and maternal manipulation in this and other facultatively social insects.

### Conclusion

Understanding how sociality may have evolved requires a firm understanding of the proximate causes of cooperation, including their physiological and behavioural basis. Our study shows that in a facultatively social bee, differences in nutritional development primarily lead to differences in gene expression associated with absolute body size, rather than future social phenotypes. Thus, the marked social differences we found in this species may be mediated by size, including associated differences in gene expression acquired during development, but likely require social context to be expressed. More generally, traits which may exist on a continuous spectrum of variation such as size are likely key to understanding social behaviour, including that in facultatively and obligately social species.

## Supplementary Material

10.1242/jexbio.250024_sup1 Supplementary information

Table S1. Head widths of mothers and head widths and pre- and post-manipulation weights, original cell position, and treatment/control group data for female brood. Weights include provisions and were made at egg or small larva stages of development. Head width was measured at eclosure/adult stage for brood, and at adult stage for mothers.

Table S2. Bees chosen for sequencing, with head widths, original cell position, and treatment/control group data. Whole heads were pulverised and sequenced using Illumina NovaSeq 6000.

Table S3. Differentially expressed genes for each contrast among control and treatment groups, including gene IDs.

Table S4. Gene ontology IDs and terms enriched in differentially expressed genes associated with each each experiemental/control group, and with bees of larger or smaller size. BP = biological process; CC = cellular compartment; MF = molecular function.

Table S5. Differentaily expressed genes associated with larger or smaller head widths/size. Log2fold change > 0 are associated with larger beest < 0 with smaller bees.

Table S6. Genes found to be correlated within supported WGCNA modules, with module color coding and number.

Table S7. Gene ontology terms and IDs enriched in genes involved in with supported WGCNA modules (Table S6).
