# Supplementary material for: Transcriptomic correlates of nutritional manipulation in a facultatively social bee
Source: J Exp Biol. 2025 Apr 16;228(8):jeb250024. doi: 10.1242/jeb.250024 (PMC12045643; doi:10.1242/jeb.250024)
Supplement: Supplementary information [file jexbio-228-250024-s1.pdf]

Table S1. Head widths of mothers and head widths and pre- and post-manipulation weights, original cell position, and treatment/control group data for female brood. Weights include provisions and were made at egg or small larva stages of development. Head width was measured at eclosure/adult stage for brood, and at adult stage for mothers.

Available for download at
https://journals.biologists.com/jeb/article-lookup/doi/10.1242/jeb.250024#supplementary-data

Table S2. Bees chosen for sequencing, with head widths, original cell position, and treatment/control group data. Whole heads were pulverised and sequenced using Illumina NovaSeq 6000.

Available for download at
https://journals.biologists.com/jeb/article-lookup/doi/10.1242/jeb.250024#supplementary-data

Table S3. Differentially expressed genes for each contrast among control and treatment groups, including gene IDs.

Available for download at
https://journals.biologists.com/jeb/article-lookup/doi/10.1242/jeb.250024#supplementary-data

Table S4. Gene ontology IDs and terms enriched in differentially expressed genes associated with each each experiemental/control group, and with bees of larger or smaller size. BP = biological process; CC = cellular compartment; MF = molecular function.

Available for download at
https://journals.biologists.com/jeb/article-lookup/doi/10.1242/jeb.250024#supplementary-data

Table S5. Differentaily expressed genes associated with larger or smaller head widths/size. Log_2 fold change > 0 are associated with larger beest < 0 with smaller bees.

Available for download at
https://journals.biologists.com/jeb/article-lookup/doi/10.1242/jeb.250024#supplementary-data

Table S6. Genes found to be correlated within supported WGCNA modules, with module color coding and number.

Available for download at
https://journals.biologists.com/jeb/article-lookup/doi/10.1242/jeb.250024#supplementary-data

Table S7. Gene ontology terms and IDs enriched in genes involved in with supported WGCNA modules (Table S6).

Available for download at
https://journals.biologists.com/jeb/article-lookup/doi/10.1242/jeb.250024#supplementary-data
